# Temporally Coordinated Deep Brain Stimulation in the Dorsal and Ventral Striatum Synergistically Enhances Associative Learning

**DOI:** 10.1038/srep18806

**Published:** 2016-01-04

**Authors:** Husam A. Katnani, Shaun R. Patel, Churl-Su Kwon, Samer Abdel-Aziz, John T. Gale, Emad N. Eskandar

**Affiliations:** 1 Department of Neurosurgery Massachusetts General Hospital Harvard Medical School, Boston, MA; 2 Department of Neuroscience Cleveland Clinic Cleveland, OH.

## Abstract

The primate brain has the remarkable ability of mapping sensory stimuli into motor behaviors that can lead to positive outcomes. We have previously shown that during the reinforcement of visual-motor behavior, activity in the caudate nucleus is correlated with the rate of learning. Moreover, phasic microstimulation in the caudate during the reinforcement period was shown to enhance associative learning, demonstrating the importance of temporal specificity to manipulate learning related changes. Here we present evidence that extends upon our previous finding by demonstrating that temporally coordinated phasic deep brain stimulation across both the nucleus accumbens and caudate can further enhance associative learning. Monkeys performed a visual-motor associative learning task and received stimulation at time points critical to learning related changes. Resulting performance revealed an enhancement in the rate, ceiling, and reaction times of learning. Stimulation of each brain region alone or at different time points did not generate the same effect.

Within the context of an associative learning task, in which visual cues dictate reaches to specific spatial locations, the ventral and dorsal sub-regions of the striatum exhibit different but complimentary roles. The ventral striatum, specifically the Nucleus Accumbens (NAc), is believed to be involved in incentive motivational processes that serve to drive goal-oriented behavior^1^,^2^. Accordingly, the neurophysiology of the NAc has been shown to have increased activation prior to the execution of a behavioral response. In contrast, the dorsal striatum, specifically the Caudate (Cd), is believed to control the selection of appropriate reward-seeking motor actions^3^. Activation patterns of the Cd have been shown to correlate with reinforced motor behavior during learning. Interestingly, phasic microstimulation in the Cd, only during the reinforcement of actions linked to positive outcome, has been shown to enhance the rate of learned associations^4^. This finding reveals that the temporal dynamics of cognitive processes unfolding during an associative learning task can be manipulated with stimulation to alter behavioral performance. Under such a framework, we asked whether we could extend the result by modifying electrophysiological patterns across multiple nodes of a network in a temporally coordinated manner using clinically relevant deep brain stimulation (DBS) in order to demonstrate a translation strategy that can improve cognitive abilities.

Here we assess the impact of DBS to alter associative learning performance when applied in a temporally precise manner across both the NAc and the Cd brain regions, respectively. The study was conducted utilizing non-human primates performing an associative learning task, in which animals were presented an abstract visual cue and were required to learn the correct spatial location associated with that cue in order to receive reward. Based on aforementioned discoveries of neural activity during associative learning, high frequency DBS was applied in the NAc at the beginning of trials and in the Cd during the reinforcement period of the task. Our results reveal that coordinated activation of the NAc and Cd during learning can enhance performance to a significantly greater extent than when activating each structure alone. Furthermore, the result could not be recreated when a different temporal strategy was applied, demonstrating the importance of precise timing in neural coordination. Taken together, the findings demonstrate the translational potential for a neuromodulatory intervention that can improve specific cognitive function.

## Materials and Methods

Experiments were performed using two adult rhesus macaque monkeys. All procedures were approved by the Massachusetts General Hospital Institutional Animal Care and Use Committee and were directed in agreement with the Public Health Services Guide for the Care and Use of Animals.

## Learning Task

**Figure 1:**
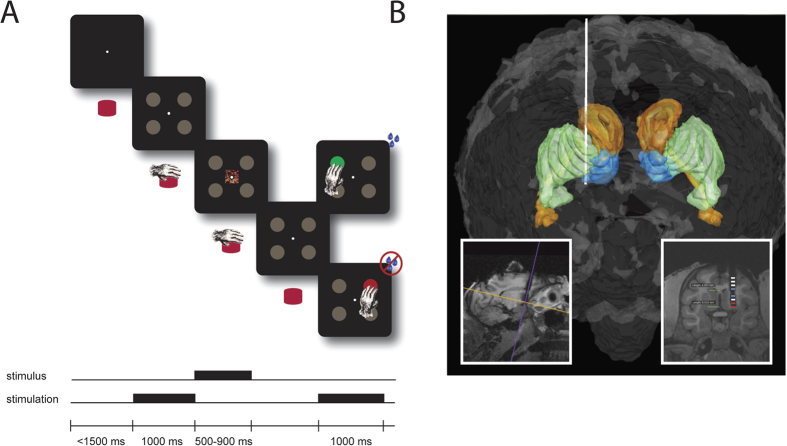
Task and Anatomy. (**A**) Spatial and temporal representation of the touch-screen based associative learning task. **(B)** 3D anatomical reconstruction using post-operative T1-MRI showing electrode placement (white lead) within segmented brain regions (putamen–green; caudate nucleus–orange; nucleus accumbens–blue). *Inset left*: Sagittal view of the MRI with cross-hairs depicting the orientation of the coronal plane. Artifact represents the signal void created by metallic components of implanted DBS lead. *Inset right*: A post-implant coronal MRI image with depiction of 8 electrode contact locations (blue–caudate, red–nucleus accumbens).

Through trial-and-error animals were required to learn associations between unique visual cues displayed at the center of a touchscreen and a reach movement to one of four peripheral targets (Fig. 1A). The beginning of each trial started with the presentation of a fixation cue. Following a brief delay, four gray targets appeared in the periphery. Using their right hand, animals were required to press and hold a home-button attached and centered on their chair. The home button controlled the starting position of the hand prior to the reach. Following a random interval, a stimulus image was presented on the center of the screen for one second. The disappearance of the image indicated to the animal to make a choice by releasing the button and touching one of the four targets. If the choice was correct, the selected target turned green and liquid reward was delivered. If the choice was incorrect, the selected target turned red and no reward was delivered.

Each stimulus image on a given block was associated with only one target location that was mutually exclusive. Presented stimulus images occurred in sets of four. Two of the images were randomly selected from a group of familiar images that the monkeys had already learned (control), and the other two from a group of new images that the monkeys had not previously seen. After an animal reached steady state learning (selected the correct target at least five times in a row) for each image, new images were associated with different target locations.

There were four different block conditions: (1) no stimulation, (2) stimulation in the nucleus accumbens at the fixation period, (3) stimulation in the caudate nucleus at the feedback period for correct choices, and (4) combined nucleus accumbens (at the fixation period) and caudate nucleus stimulation (during feedback on correct trials). In addition, only one of the novel stimulus images received stimulation for each stimulation block. The other novel image remained as an internal control. On a daily session the order of the four blocks was randomly assigned and selected without replacement. On average each animal played all four blocks each day (animal 1: mean = 4.38 blocks, std: 2.09; animal 2: mean = 3.11 blocks, std: 1.26) All behavioral features of the task (i.e., reaction time, target touched) were captured and stored for off-line analysis using Monkeylogic^5^.

## Deep Brain Stimulation

Primates were implanted with a commercially available customized miniaturized DBS lead (NeuMed; Trento NJ). The lead consisted of a 16 cm polymer tubing with a diameter of 1.27 mm and eight concentric platinum iridium (90/10) contacts. The eight electrodes were grouped into two sets of four contacts with 4.5 mm spacing between each group. The four most distal contacts, spaced 0.5 mm apart, were aimed to target the nucleus accumbens and the four more proximal contacts, spaced 1 mm apart, aimed to target the caudaute nucleus. The caudate nucleus resides just above the accumbens in the dorsal/ventral axis allowing both nuclei to be stereotactically targeted with one lead trajectory. Prior to surgery animals received a pre-operative MRI with a fiducial marker grid in order to determine the best stereotactic coordinates for targeting the striatum. Post-operative MRI was then used to confirm the placement of each electrode with respect to the striatum. In addition, each slice of the MRI was imported into MATLAB to render a 3D reconstruction of the MRI with overlaying brain region boundaries (Fig. 1B). Based on imaging, the implant procedure for both animals resulted in electrode contacts that resided approximately in the anterior-lateral portion of the head of the caudate nucleus and the lateral portion of the nucleus accumbens shell. Two contacts in each set of four were selected to be the source and sink for bipolar DBS in these regions of the caudate (contact 3+, 5−) and accumbens (contact 1+, 2−), respectively. Based on imaging, the contact that resided most directly in the anterior-lateral portion of the head of the caudate nucleus and the lateral portion of the nucleus accumbens shell was set as the anode. Constant current stimulation was delivered via a DS8000 stimulator in combination with DSL1000 isolation units (World Precision Instruments, Sarasota, FL, USA). Stimulation parameters set to deliver phasic stimulation in each nuclei were the same and delivered a charge balanced biphasic stimulating pulse, anodic leading, with an amplitude of 1 mA, frequency of 200 Hz, total pulse width of 0.4 ms, and for a duration of 1000 ms. Only the onset timing of stimulation differed between the caudate and accumbens (Fig. 1A). Commercially available DBS systems have upper limits of 25 mA and 250 Hz and allow for a maximum charge density 30 μC/cm^2^/phase. The selected parameters for this study generate a total charge density of 0.008 μC/cm^2^/phase, falling well below the safe threshold of clinical DBS systems.

## State-Space Model

We utilized a previously established state-space model to characterize the probability of a correct response as a function of trial number for each block condition, respectively^6^. The model outputs calculated learning curves and a learning criteria trial that describes the estimated occurrence of learning for each curve. The learning criteria is defined as the first trial in which the lower 95th percentile confidence bound exceeds chance. Chance level for each block condition was defined by the mean performance of the first trial.

## Data Analysis

All distributions passed tests for normality (Kolmogorov-Smirnov) and for equal variance (Levene Median), unless noted differently. Learning curves generated from each block were tested against one another with a repeated measure two-tailed t-test to reveal differences between trials. Reaction time distributions were tested with a two-tailed Students t-test. The learning criteria distributions output from the state space model did not pass normality. Accordingly, we applied a non-parametric test (Wilcox Rank Sum). Data from each animal was tested separately.

## Results

### Effects of deep brain stimulation on learning behavior

**Figure 2:**
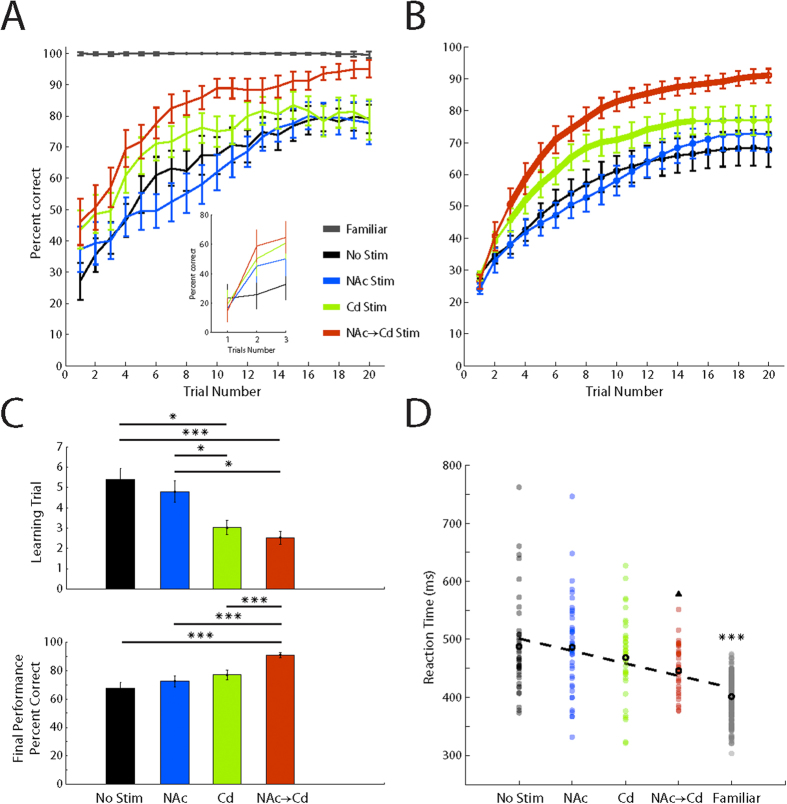
Effects of Deep Brain Stimulation on Associative Learning. (**A**) Learning curves conveyed as percent correct across trials from animal 1. Traces represent a moving average (window size = 4) of the correct and incorrect choices made by the animal for each block condition. No Stim block (black trace) composed of n = 43 blocks (animal 2: n = 48 blocks). NAc Stim block (blue trace) composed of n = 42 blocks (animal 2: n = 25 blocks). Cd Stim block (green trace) composed of n = 39 session (animal 2: n = 27 blocks). NAc → Cd Stim block (red trace) composed of n = 34 blocks (animal 2: n = 20 blocks). Familiar images from all block conditions (gray trace) composed of n = 158 blocks (animal 2: n = 120 blocks). *Inset:* Mean percent correct for each of the first three trials (no sliding window). (**B**) State-space approach learning curves for each block condition from animal 1. Thick areas along traces indicate trials where performance on stimulated trials was significantly different from performance on non-stimulated trials. (**C**) *Top:* Distribution of learning criteria for each block condition. *Bottom:* Final performance for each block condition. (**D**) Scatter plot of reaction time sorted by final performance of each block condition and familiar images. Thick black circles represent the mean for each distribution. Dashed line represents a linear regression fit to the mean reaction time for each distribution. Triangle (▲) signifies significant difference of the NAc → Cd Stim block from the No Stim, NAc Stim, and Cd Stim blocks. Asterisk (*) signifies significant difference of the Familiar block from the No Stim, NAc Stim, Cd Stim, and NAc → Cd Stim blocks. All values are mean +/− s.e.m. (▲)*p* < 0.05, ***p < 0.001.

**Figure 3:**
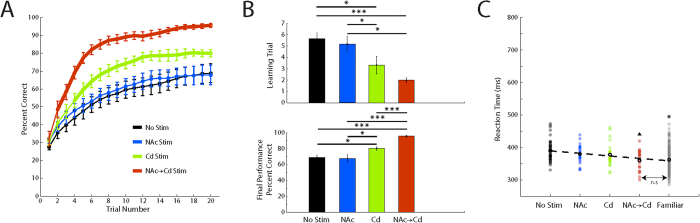
Effects of Deep Brain Stimulation on Associative Learning. (**A**) State-space approach learning curves for each block condition from animal 2. Thick areas along traces indicate trials where performance on stimulated trials was significantly different from performance on non-stimulated trials. (**B**) *Top:* Distribution of learning criteria for each block condition. *Bottom:* Final performance for each block condition. (**C**) Scatter plot of reaction time sorted by final performance of each block condition and familiar images. Thick black circles represent the mean for each distribution. Dashed line represents a linear regression fit to the mean reaction time for each distribution. Triangle (▲) signifies significant difference of the NAc → Cd Stim block from the No Stim, NAc Stim, and Cd Stim blocks. Asterisk (*) signifies significant difference of the Familiar block from the No Stim, NAc Stim, and Cd Stim blocks. All values are mean +/− s.e.m. *(▲)*p* < 0.05, n.s – not significant.

We trained two animals to perform a touch-based visual-motor associative learning task. We first computed learning curves by applying a sliding window average across correct and incorrect choices in order to observe the shape of learning curves generated from each block condition (Fig. 2A). We confirmed that the animals performed very well for familiar images pooled from each block (>98% across all trials). Next, we evaluated novel images from block condition 1 (No Stim) and only novel images that were stimulated for block condition 2 (NAc Stim), 3 (Cd Stim) and 4 (NAc → Cd Stim). For these novel images, we observed a trend that followed a logistic function, in which the mean start performance for each condition on trial 1 was ~25% (Fig. 2A, inset) with the ceiling for learning at ~79% for the No Stim, NAc Stim and Cd Stim blocks, and the ceiling for NAc → Cd Stim block at ~95%. As a next step to quantify characteristics of learning, we implemented a state-space approach, which is utilized to fit binomial distributions with a logistic function for full details^6^. Figure 2B illustrates the average learning curves estimates from the model. Similar to a previous finding by Williams *et al.* (2006) we show that the animals have a significant increase in the rate of rise (two-tailed t-test, incremented comparison, *p* < 0.05) for learning performance when receiving stimulation in the Cd during the feedback epoch as compared with the No Stim condition learning curve (Figs 2B and 3A, thick green trace). In line with this, the learning curves significantly separate in as little as three trials. Importantly, with the addition of NAc stimulation at the beginning of trials we found that animals not only increased in the rate of rise (two-tailed t-test, incremented comparison, *p* < 0.001), also separating at the third trial, but also that the ceiling for learning was increased (Figs 2B and 3A, thick red trace). No differences were found between learning curves from the No Stim and NAc Stim blocks. To further quantify these findings we evaluated the learning criteria trial, a proxy for learning rate, output by the model as well as the final performance for each block condition.

### Changes in learning curve characteristics and response times

The learning criteria (Figs 2C and 3B, top) was identified as the trial in which the lower 95 confidence interval of the logistic function exceeded chance level. Learning criteria distributions for each of the four block conditions were tested against the three remaining distributions separately. Animals reached the learning criteria in significantly fewer trials during the Cd Stim and NAc → Cd Stim blocks (rank sum test, *p* < 0.05) when compared to No Stim and NAc Stim blocks. Although there was no significant difference between the Cd Stim and NAc → Cd Stim blocks (rank sum test, *p* = 0.28), the learning trial for the NAc → Cd block tended to occur earlier, indicating that the addition of stimulation in the NAc at the beginning of trials further boosted learning performance. Final performance (Figs 2C and 3B, bottom) was defined as the average percent correct on the last trial for each block condition. In one animal we found no significant difference in the final performance (two-tailed t-test, *p* >* *0.05) between the No Stim, NAc Stim and Cd Stim blocks. The second animal had a greater final performance for the Cd Stim block (two-tailed t-test, *p* < 0.05). Importantly, however, both animals had a significantly higher final performance (two-tailed t-test, *p* < 0.001) for the NAc → Cd block when compared to each block separately, revealing that the combination of precisely timed phasic NAc and Cd stimulation can have a synergistic effect to improve final performance.

To examine the possible role of stimulation on reaction time, we examined the distributions for each of the four block conditions. To visualize this we sorted the blocks by mean final performance and scattered the reaction times (Figs 2D and 3C). We observed a negative correlation (animal 1: *β* = −21.43, *p* <* *0.001, animal 2: *β* = −7.71, *p* < 0.001) between mean reaction time and the sorted block conditions. Additionally, reaction times for familiar images were significantly shorter (two-tailed t-test, *p* < 0.001) when compared to reaction times for novel images in the No Stim, NAc Stim and Cd Stim blocks. Similarly, reaction times from the NAc → Cd block were also significantly shorter (two-tailed t-test, *p* < 0.05) when compared to each block condition. Interestingly, in one animal there was no statistical difference (two-tailed t-test, *p* = 0.81) between the reaction times distributions from the NAc → Cd block and from familiar images (Fig. 3C). Taken together these findings suggest that NAc → Cd stimulation allowed animals to consistently perform faster and to an operational proficiency closer to that of the performance seen on familiar images.

### Controls for association selectivity, reward and temporal specificity

**Figure 4:**
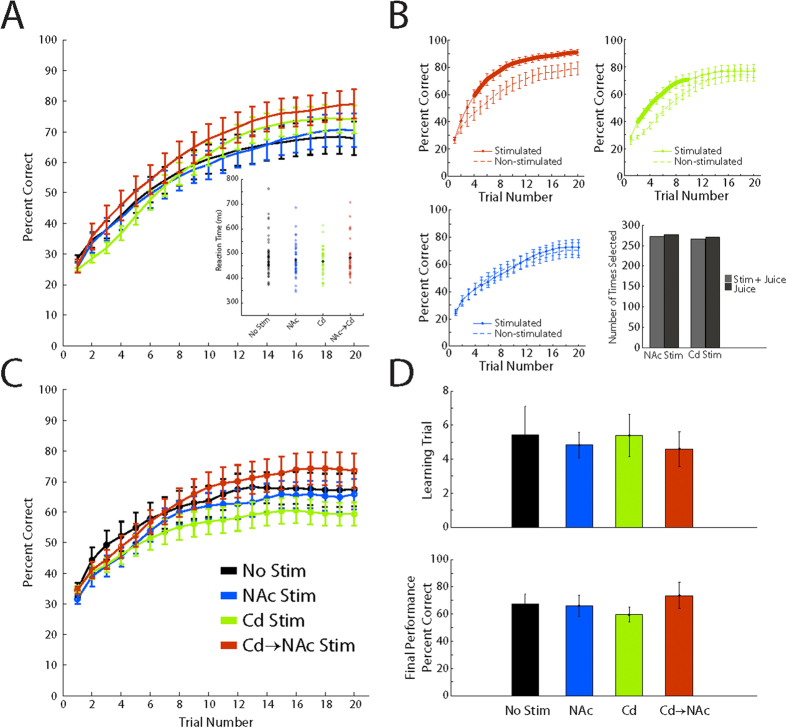
Stimulation Control Experiments. **(A)** State-space approach learning curve estimates for the non-stimulated novel image for each block condition from animal 1. Color scheme maintained from Figure 1. *Inset:* Distribution of reaction times for each block condition. Thick black circles represent the mean for each distribution. (**B**) *top and bottom left,* State-space approach learning curves for stimulated (solid line) and non-stimulated (dashed) images within the NAc → Cd Stim blocks (red traces), Cd Stim blocks (green traces), and NAc Stim blocks (blue traces). Thick areas along traces indicate trials where performance was significantly different. ***bottom right***, Count of target choice for hedonic control test for both NAc and Cd stimulation in animal 1. Light gray bar represents choice of juice reward with stimulation (animal 2: NAc = 252, Cd = 263). Dark gray represents choice of just juice reward (animal 2: NAc = 265, Cd = 247). (**C**) State-space approach learning curves for block conditions from animal 1, in which the epoch for NAc and Cd stimulation were reversed. No Stim block (black trace) composed of n = 31 blocks (animal 2: n = 12 blocks). NAc Stim block (blue trace) composed of n = 12 blocks (animal 2: n = 8 blocks). Cd Stim block (green trace) composed of n = 15 session (animal 2: n = 10 blocks). Cd → NAc Stim block (red trace) composed of n = 7 blocks (animal 2: n = 7 blocks). (**D**) *Top:* Distribution of learning criteria for each block condition of the reverse epoch stimulation control. *Bottom:* Final performance for each block condition of the reverse epoch stimulation control. All values are mean +/− s.e.m.

Inherent to our task design was a novel image learned concurrently with stimulated images. The image acted as a control to test response bias and provided information as to the specificity of the stimulation effect. The state-space model analysis (Fig. 4A) on the performance for these images revealed that there were no significant differences across trials (two-tailed t-test, incremented comparison, *p* >* 0.05*), in the learning rate (rank sum test, *p* >* *0.1) or in the final performance (two-tailed t-test, *p* >* *0.5) across all block conditions, as compared to baseline No Stim trials. In addition, there were no significant differences (two-tailed t-test, *p* >* *0.2) in the reaction time distributions (Fig. 4A, inset). These results indicate that the improvement in learning was not attributed to a nonspecific directional bias toward the stimulated target location. Importantly, a direct comparison of learning on non-stimulated and stimulated novel images that were presented in the Cd Stim and NAc → Cd Stim blocks revealed that learning curves significantly separated (two-tailed t-test, incremented comparison, *p* < 0.001) in a similar manner as when compared to learning during the No Stim blocks (Fig. 4B, top, thick red and green traces). The finding demonstrates that the effect of stimulation was selective for specific associations.

We also asked whether stimulation-induced activity in the NAc was perceived as a pleasurable or rewarding experience to the animals. The effect of NAc stimulation may produce a hedonic response bias that leads the animal to favor a particular motor response. Accordingly, we conducted a control experiment, in which animals selected between two visually identical targets. Both targets resulted in a liquid reward with one target also resulting in concomitant stimulation in the NAc. The location of the target on the screen was randomized each day. Working under the premise that stimulation in the NAc does produce a hedonic experience, one would expect the animal to predominantly choose the target associated with both reward and stimulation. In contrast to this hypothesis, we found no difference in the animals’ choice between targets (Fig. 4B), demonstrating that stimulation did not generate a hedonic response. Instead, the finding suggests the stimulation is acting to augment an intrinsic process implicated in image-response associations. Interestingly, this finding contrast with previous studies that have utilized microstimulation in the NAc to alter motivation and choice behavior^7^,^8^. We attribute the difference in result to the localization of the electrode, from the accumbens core to the shell, as well as the utilization of a DBS electrode. Given the relatively large surface area of DBS electrode contacts, the resulting charge density will be low compared to that generated by a high impedance micro-electrode^9^. As a result, expectations based on previous findings may not translate directly to this control experiment.

To address the question as to whether the temporal specificity of stimulation played an important role in the synergistic enhancement of associative learning, we conducted a control experiment in which the temporal delivery of NAc and Cd stimulation was reversed. Accordingly, four different block conditions were maintained: (1) No stimulation, (2) Cd stimulation delivered at the fixation period, (3) NAc stimulation delivered during the feedback epoch of correct outcomes and (4) Cd stimulation delivered at the fixation period followed by NAc stimulation delivered during the feedback epoch of correct outcomes. Statistical analysis on the state-space model output (Fig. 4C,D) revealed that there were no significant differences across trials (two-tailed t-test, incremented comparison, *p* >* *0.05), in the learning rate (rank sum test, *p* >* *0.5) or in the final performance across (two-tailed t-test, *p* >* *0.1) across all block conditions, signifying the importance of delivering stimulation in time periods relevant to learning related changes.

## Discussion

Previous findings from neurophysiological studies in non-human primates have suggested that the Cd and NAc are integrally involved in associative learning^10^,^11^, but at distinct task-epochs^4^,^12^. From these results, we posited that the application of coordinated electrical stimulation following the observed temporal specificity could multiplicatively enhance learning. In this study, we demonstrate that a burst of stimulation in the NAc at the beginning of a learning trial combined with a burst of stimulation in the Cd during the reinforcement period of positive outcomes can significantly enhance learning performance above baseline performance. Empirically, animals took fewer trials to learn a given visual-motor association and reached a final performance nearing 95% correct. This outcome is surprising when compared to the baseline performance that took nearly double the number of trials to reach learning criteria and with a final proficiency near 80% correct. Even more impressive was the fact that the mean and standard deviation of reaction time distributions were reduced, emulating response time characteristics seen on familiar images, and suggesting that coordinated stimulation allowed for a reduction in uncertainty and thus a higher proficiency. Interestingly, when stimulation was confined to a single brain region, the observed performance effects vanished. Stimulation in the NAc at the beginning of a trial did not alter performance and although stimulation in the Cd during the reinforcement period increased the learning rate, there was no effect on the asymptote for performance or the response times. When a different temporal strategy was utilized, in which the epoch for stimulation delivery was switched, no performance effects were observed. Taken together, these findings show that only when stimulation of each region was combined, with a temporally specificity relevant to learning dynamics, did a synergy occur to maximize learning.

Changes in the learning performance in this study occurred on a relatively fast time-scale, with the learning criteria being met in about 5 trials from the start of a session and animals reaching a steady state for final performance in less than 20 trials. The ability for stimulation to rapidly modulate neural circuitry to affect fast behavioral dynamics suggests a neural mechanism with a short time-constant. Although the precise mechanism is not known, neurophysiological findings support the notion that the intrinsic dynamics of neurotransmitter release for the dopaminergic system may underlie the observed learning enhancements found in this study^13^,^14^. The midbrain dopaminergic system has been shown to play a critical role in learning and goal directed processes through phasic striatal dopamine release^15^. The Cd has input-output zones^16^,^17^ with specific dopaminergic afferents from areas such as the substantia nigra pars compacta (SNC) that can allow for the potentiation of corticostriatal synapses involved in motor and association skills^18^. Neural activity in the caudate nucleus has been shown to follow the learning rate^4^, suggesting a role for dopamine induced reinforcement learning^19^. Additionally, stimulation during this period enhances the learning rate, likely through phasic dopamine release^20^.

Of interest to this study, however, is how stimulation of the NAc at the beginning of a learning trial can be compounded with stimulation in the Cd that occurs seconds later. A potential clue might be in examining timescales. The dopamine system and its effects on learning are classically studied on short timescales, that is, phasic dopamine release has been proposed to initiate behaviors^21^,^22^ and encode prediction error signals^19^,^23^. However, recently, evidence from Howe *et al.*^24^ has suggested that information is also represented over longer timescales. Similarly, neurophysiological evidence from the primate NAc has shown progressively increasing activity relative to learning over long timescales^12^. In addition, electrical stimulation of the NAc in primates acts to increase task engagement, even in the absence of primary rewards, ostensibly by increasing motivation or incentive salience^8^. Furthermore, the dopaminergic connections of the ventral striatum have been shown to be widespread and extensive, having influence on many areas of the dopaminergic system including the SNC^25^. We speculate that this influence could provide the NAc with the capability to affect the dopamine inputs of the Cd. As a result, stimulation of NAc at the beginning of a trial could energize the next response by enhancing the long time-scale evolution of motivational state processing^26^. Accordingly, this effect could compound with the reinforcement learning processes of the Cd to further enhance execution of the current goal^27^. We provide this description only as speculation and of course alternative hypotheses may also exist. For example, there is converging evidence to suggest that striatal stimulation can induce behavioral change by modulating long-term potentiation of learning networks, inducing neural plasticity, and altering oscillatory firing patterns to change global brain states^1^,^28^,^29^,^30^. Each potential mechanism can stand as a mechanistic underpinning for the observed enhancement in associative learning. It is also important to note that there are different learning strategies that can be employed for the same learning task. For example, stimulation may selectively increase the salience of the stimulated images, allowing animals to better discriminate against competing images. Future experiments must focus on how stimulation can affect learning within different paradigms in order to better understand the properties of the intervention in the context of enhancing learning.

Deep brain stimulation (DBS) is a powerful method by which to interface with the brain. In its current state, DBS is typically confined to a single brain region, in which stimulation is applied in a continuous open-loop fashion. Through this approach, DBS has proved to be an effective treatment strategy for Parkinson’s disease^31^ and is actively being explored for other indications^32^. As the technology for DBS advances, studies utilizing implantable stimulation devices are presenting more sophisticated approaches. Devices are now capturing neural activity in real-time in order to deliver stimulation at relevant time points that better match the intrinsic neurophysiology within a targeted brain region. Accordingly, we argue that the multi-scale encoding of the brain supports a rationale for a temporally distinct coordinated electrical stimulation approach, which may augment intrinsic mechanisms in a synergistic fashion to maximize behavioral outcomes. A rational of this nature can foreseeably translate to helping patients with neurological deficits. Specific to this investigation, enhancing visuo-motor performance may be beneficial to patients suffering from cognitive-motor deficits caused by traumatic brain injury. Furthermore, a DBS strategy that can enhance learning, memory and motivational processes could be used in humans to enhance a broad range of functions including gross motor movements, decision making, and certain sensory impairments. Theoretically any particular set of associations could be reinforced to augment learning and improve functional outcomes.

## Additional Information

**How to cite this article**: Katnani, H. A. *et al.* Temporally Coordinated Deep Brain Stimulation in the Dorsal and Ventral Striatum Synergistically Enhances Associative Learning. *Sci. Rep.* **6**, 18806; doi: 10.1038/srep18806 (2016).
